# Aberrant DNA methylation and overexpression of NR0B1 are prognostic biomarkers in KEAP1-mutant lung adenocarcinomas

**DOI:** 10.1007/s12672-025-03484-1

**Published:** 2025-08-27

**Authors:** Mohamed Elshaer, Ahmed Hammad, Yang Gao, Xiu Jun Wang, Xiuwen Tang

**Affiliations:** 1 Department of Respiratory and Critical Care Medicine, Center for Oncology Medicine, International School of Medicine, The Fourth Affiliated Hospital of School of Medicine, International Institutes of Medicine, Zhejiang University, Yiwu, 322000 China; 2 Department of Biochemistry, Zhejiang University School of Medicine, Hangzhou, 310058 People’s Republic of China; 3 Department of Pharmacology and Cancer Institute, The Second Affiliated Hospital, Zhejiang University School of Medicine, Hangzhou, 310009 People’s Republic of China; 4 Labeled Compounds Department, Hot Labs Center, Egyptian Atomic Energy Authority, Cairo, 13759 Egypt; 5 Department of Biochemistry and Molecular Biology, Medical University of South Carolina, Charleston, SC 29425 USA; 6 Radiation Biology Department, National Center for Radiation Research and Technology, Egyptian Atomic Energy Authority, Cairo, 13759 Egypt

**Keywords:** KEAP1, NRF2, Lung cancer, DNA methylation, NR0B1

## Abstract

The KEAP1-NRF2 pathway regulates cytoprotective responses to endogenous and exogenous stresses induced by reactive oxygen species (ROS) and electrophiles. To maintain oxidative homeostasis, cancer cells increase the transcription of antioxidant genes by acquiring either stabilizing mutations in *NRF2* or inactivating mutations in its negative regulator, *KEAP1. NR0B1* is a recently identified NRF2 target that supports anchorage-independent growth in *KEAP1*-mutant non-small cell lung cancer (NSCLC) cells. In this study, we used multi-omics and wet-lab approaches to explore the correlation between *KEAP1* mutations, the methylation status of the *NR0B1* promoter, *NR0B1* gene expression and survival of patients with lung adenocarcinoma (LUAD). We found that the NR0B1 promoter was highly hypomethylated and NR0B1 was significantly overexpressed in KEAP1-mutant LUAD cell lines and patient samples compared to their wild-type counterparts. Correlation analysis showed a strong negative correlation between the hypomethylated CpG sites (*NR0B1* promoter) in *KEAP1* mutant LUAD patients and NR0B1 gene expression. Moreover, hypomethylation of cg22696549 CpG site (*NR0B1* promoter) was associated with poor survival in LUAD patients. Furthermore, the overexpression of *NR0B1* was correlated with worse prognosis of LUAD patients. These findings suggest a potential synergy between *KEAP1* mutation-associated DNA methylation changes and the transcription factor NRF2, which may drive *NR0B1* overexpression. Additionally, this study may enhance the understanding of the molecular mechanisms associated with NR0B1 in lung cancer progression and drug resistance.

**Supplementary Information:**

The online version contains supplementary material available at 10.1007/s12672-025-03484-1.

## Introduction

Cancer cells undergo metabolic reprogramming to maintain a steady sources of energy and building blocks for cell division and rapid growth [1]. This leads to the generation of toxic metabolic byproducts, including reactive oxygen species (ROS) that can induce oxidative stress and inhibit cancer cell viability and growth [2]. This oxidative stress arising from the excessive energy and material demands of cancer cells is mainly counteracted via activation of the nuclear factor erythroid 2-related factor 2 (NRF2) pathway, a master regulator of the cellular antioxidant response [3, 4]. Under basal conditions, the basic leucine zipper (bZip) transcription factor NRF2 binds to its negative regulator kelch-like ECH-associated protein 1 (KEAP1), which then mediates rapid and constitutive ubiquitination and proteasomal degradation of NRF2 [5]. Under oxidative stress, key cysteine residues in KEAP1 are oxidatively modified to block interactions with NRF2, stabilizing the transcription factor, enabling its nuclear translocation. This results in the coordination of a gene expression program that induces detoxification and metabolic enzymes to restore redox homeostasis [2]. Various types of cancer enhance NRF2 function in multiple ways, including genetic mutations in *KEAP1* that disrupt their interaction and are found in 17% of lung adenocarcinoma (LUAD) cases [6].

The orphan nuclear receptor *NR0B1* is a recently identified NRF2 target that has been shown to support the anchorage-independent growth of *KEAP1*-mutant non-small cell lung cancer (NSCLC) cells. It forms a multimeric transcriptional complex important for regulating a subset of the NRF2 gene expression program in NSCLC cells [7]. Moreover, it has been demonstrated that targeting the conserved cysteine (C274) residue of NR0B1 disrupts NR0B1 protein complexes, altering the transcriptional output and growth of NRF2-activated cancer cells [7]. In our previous study, we showed that the DNA of several well-known NRF2 targets is hypomethylated in *KEAP1*-mutant LUAD patient samples compared to their wild-type counterparts. This finding is strongly correlated with their over-expression in *KEAP1*-mutant patient samples [8]. In the present study, we applied multi-omics and wet lab approaches to investigate the DNA methylation status of *NR0B1* in *KEAP1*-mutant LUAD cell lines and patient samples compared to their wild-type counterparts. In addition, we investigated the correlation between these DNA methylation changes and *NR0B1* gene expression. Interestingly, we identified a CpG island located 200 bp upstream of the transcription start site (TSS) in the promoter of *NR0B1*, which was hypomethylated in both *KEAP1*-mutant LUAD cell lines and patient samples. In addition, we found that the hypomethylation of the cg22696549 CpG site (200 bp upstream of the TSS in the *NR0B1* promoter) was associated with poor survival of LUAD patients. Furthermore, high *NR0B1* gene expression was correlated with worse survival outcomes in patients with LUAD (Table 1).

## Methods

### Cancer Cell Line Encyclopedia (CCLE) data analysis

RNA-seq gene expression and DNA methylation data for 1019 cell lines were downloaded from CCLE (https://portals.broadinstitute.org/ccle/data). We identified LUAD cell lines that harbor *KEAP1* mutations using the COSMIC database (https://cancer.sanger.ac.uk/cell_lines). Then, we divided the LUAD cell lines into two groups *KEAP1*-mutant and wild-type (neither *KEAP1* nor *NRF2* mutations). Then, Differential DNA methylation and gene expression analyses were carried out between *KEAP1*-mutant and wild-type cell lines groups as described in detail later in the Methods section.

**Table 1:** Clinical features of the included data set

Clinical features	Statistical results
Total number of patients	478
*Gender*	
Male Female	218 (45.6%) 260 (54.4%)
*Age*	
< 60 ≥ 60	141 (28.9%) 337 (70.5%)
*History of smoking*	
Yes No	409 (85.5%) 69 (14.4%)
*Tumor stage*	
I	260 (54.4%)
II	111 (23.2%)
III	75 (15.7%)
IV Not reported	24 (5.1%) 8 (1.6%)
*TNM (AJCC) stage* *T stage*	
T1	161 (33.6%)
T2	255 (53.4%)
T3	45 (9.4%)
T4	13 (2.7%)
Tx	4 (0.8%)
*N stage*	
N0	311 (65%)
N1	86 (17.99%)
N2	67 (14.1%)
N3	1 (0.2%)
Nx	13 (2.7%)
*M stage*	
M0	312 (65.2%)
M1	24 (4.9%)
Mx	142(29.7)

### Reagents and cell culture

Unless otherwise stated, all reagents were from Sigma–Aldrich Co., Ltd. (St. Louis, USA), and all antibodies were from Santa Cruz Biotechnology (Dallas, USA). A549 and Calu3 (NSCLC) cell lines were obtained from the American Type Culture Collection (Manassas, USA). All cells were cultured at 37 °C in 95% air and 5% CO2, and passaged every 3–4 days [9]. All media supplements for cell cultures were from Invitrogen (Carlsbad, USA).

### Western blot

Proteins were denatured in Laemmli buffer containing 5% 2-mercaptoethanol for 5 min at 100 °C [10]. Cell lysates along with pre-Stained Protein Ladder marker were separated by discontinuous sodium dodecyl sulfate and a 10% polyacrylamide gel electrophoresis (SDS-PAGE). Proteins were transferred to PVDF membranes using a blotting device at 63 V on ice for 3 h and membranes were blocked for 1 h in 10% Non-fat milk solution. The blots were incubated with primary antibodies against NRF2 (1:2500), NR0B1 (1:1000), and beta-actin (1:5000) overnight at 4 °C followed by washing with 1X PBST buffer. Secondary peroxidase-conjugated antibody was used at a dilution of 1:5,000. The membrane was then analyzed using a Bio-rad scanner.

### DNA extraction

Genomic DNA was extracted from A549 and Calu3 cell lines using the Epitect^®^ lyseALL lysis kit (Qiagen, Hilden, Germany) according to the manufacturer’s instructions. DNA quality and concentration were assessed using the NanoDrop 2000 Spectrophotometer (Thermo Scientific, USA).

### Bisulfite conversion

Bisulfite conversion of genomic DNA from cell lines was performed using the Epitect^®^ Fast DNA Bisulfite kit (Qiagen, Hilden, Germany), according to the manufacturer’s instructions. Treatment with sodium bisulfite converts unmethylated cytosine residues into uracil residues, whereas methylated cytosines remain unchanged under the same conditions [11]. For amplification of the differentially methylated region of *NR0B1* gene promoter, the primers sequences were as follow: *NR0B1* forward primer, 5′-AATGGAAAGAGTTGTAATAGTATTTAGG- 3′ and *NR0B1* reverse primer, 5′-CTACCTCCTAAAACCTATTTATACCTAAC-3′. In PCR tubes, a reaction mixture (20 µl) was prepared which included 1x GC buffer I (TAKARA, Kusatsu, Japan), 2.5 mM Mg^2+^, 0.2 mM dNTPs, 0.2 µM of each primer, 1 U HotStarTaq polymerase (TAKARA, Kusatsu, Japan) and 1 µl template DNA. The PCR tubes were placed in thermal cycler and the cycler conditions was set as follow: 1 cycle of denaturation (2 min at 95 °C); 11 cycles of (20 s at 94 °C, 40 s at 62 °C (−0.5 °C/cycle), 1 min at 72 °C); 24 cycle of (20 s at 94 °C, 30 s at 56 °C, 72 °C 1 min); 1 cycle of 2 min at 72 °C; and finally hold at 4 °C. The PCR products were subjected to agarose gel electrophoresis alongside a DNA size marker.

###  Purification of PCR products using SAP and Exo I

0.5 U SAP and 4 U Exo I (Waltham, USA) were added to 8 µl of PCR products. The mixture was incubated at 37 °C for 60 min, followed by incubation at 75 °C for 15 min.

### Sequencing reaction

#### Sequencing primers

*NR0B1*_R CTACCTCCTAAAACCTATTTATACCTAAC: Reaction mixture included 3 µl BigDye3.1 mix, 2 µl sequencing primer (1 µM) and 1–2 µl purified PCR product. The cycling program was 96 °C 1 min; 28 cycles (96 °C 10 s, 50 °C 5 s, 60 °C 4 min); hold at 4 °C. The sequencing products were put into the ABI3730XL sequencer, the sequencing files were analyzed with Polyphred software, and the results were sorted out after combining with manual proofreading and recording [12]. The ratio of C peak to T peak was used to determine the level of unmethylation, complete methylation or different degree of methylation of CpG dinucleotide. According to the methylation status of CpG dinucleotide, each CpG dinucleotide was assigned as: unmethylated: 0; different degrees of methylation: < 33% = 1, 33 ~ 67% = 2, >67% = 3, and Complete methylation = 4.

### DNA methylation analysis of TCGA-LUAD patients

TCGA-LUAD genome-wide methylation data (level 3) that were generated using the Illumina Infinium Human DNA Methylation 450 K array platform was downloaded using the Broad GDAC (Global Data Assembly Centers) Firehose website (http://gdac.broadinstitute.org/). Differential DNA methylation analysis between the *KEAP1*-mutant (83 patients) and the wild-type (395 patients) groups was carried out by calculating the delta β-value for all the probes between the different groups. We identified differentially methylated CpG sites using a two-tailed unpaired Student’s t test and *p* value was adjusted using FDR method and FDR < 0.05 was considered significant, and we used a delta β-value cutoff of >|0.2|.

### RNA-Seq data analysis of TCGA-LUAD patients

TCGA RNA-Seq gene expression version 2 level 3 data (Illumina Hiseq platform) were downloaded and 478 LUAD tissues were divided into two groups: *KEAP1*-mutant (83 patients) and wild-type (395 patients) groups and subjected to differential gene expression analysis. Briefly, level 3 transcriptomic data were normalized by the RSEM method. All gene expression values were log-transformed to approximate the data to a normal distribution. Further, genes with zero values in more than 25% of the patients were excluded. The differentially expressed genes (DEGs) were identified by applying a two-tailed unpaired t-test assuming unequal variance. Then, *P* values were adjusted using the FDR method. DEGs with FDR $\:<\:$0.05 were considered significant.        

###  Correlation analysis of DNA methylation and gene expression 

A total of 77 LUAD patient samples (26 *KEAP1*-mutant and 51 wild-type) were used to detect significant correlations. The correlation test statistic was based on Pearson’s correlation coefficient between DNA methylation beta values and gene expression levels.

### Survival analysis

The prognostic value of the differentially-methylated CpG sites in *KEAP1*-mutant LUAD patient samples was assessed by analyzing the overall survival in the TCGA-LUAD cohort using the Methsurv database (https://biit.cs.ut.ee/methsurv/) [13]. A total of 461 patient samples were divided into two groups. The first group included LUAD patients in whom these CpG sites were hypomethylated (lower group) while the second group included LUAD patients in whom these CpG sites were hypermethylated CpG sites (higher group). Moreover, we evaluated the prognostic value of NR0B1 gene expression in LUAD using the Kaplan–Meier Plotter database, which includes survival data from 2852 lung cancer tumor specimens across 17 independent cohorts, of which 1161 cases (55%) are lung adenocarcinomas (LUAD). The survival analysis was conducted using all available LUAD patients (*n* = 1,161). As representative examples, we cite the following cohorts: (GSE14814) (*n* = 27) [14], (GSE19188) (*n* = 40) [15], (GSE29013) (*n* = 30) [16], (GSE30219) (*n* = 85) [17], (GSE31210) (*n* = 226) [18], (GSE3141) (*n* = 58) [19], (GSE37745) (*n* = 106) [20], and (GSE50081) (*n* = 127) [21]. Survival analysis was performed using the Kaplan–Meier Plotter online tool (http://kmplot.com/analysis/index.php%3Fp=service%26cancer=lung) [22].

## Results

### NR0B1 is hypomethylated and overexpressed in KEAP1-mutant LUAD cell lines

**Fig. 1:**
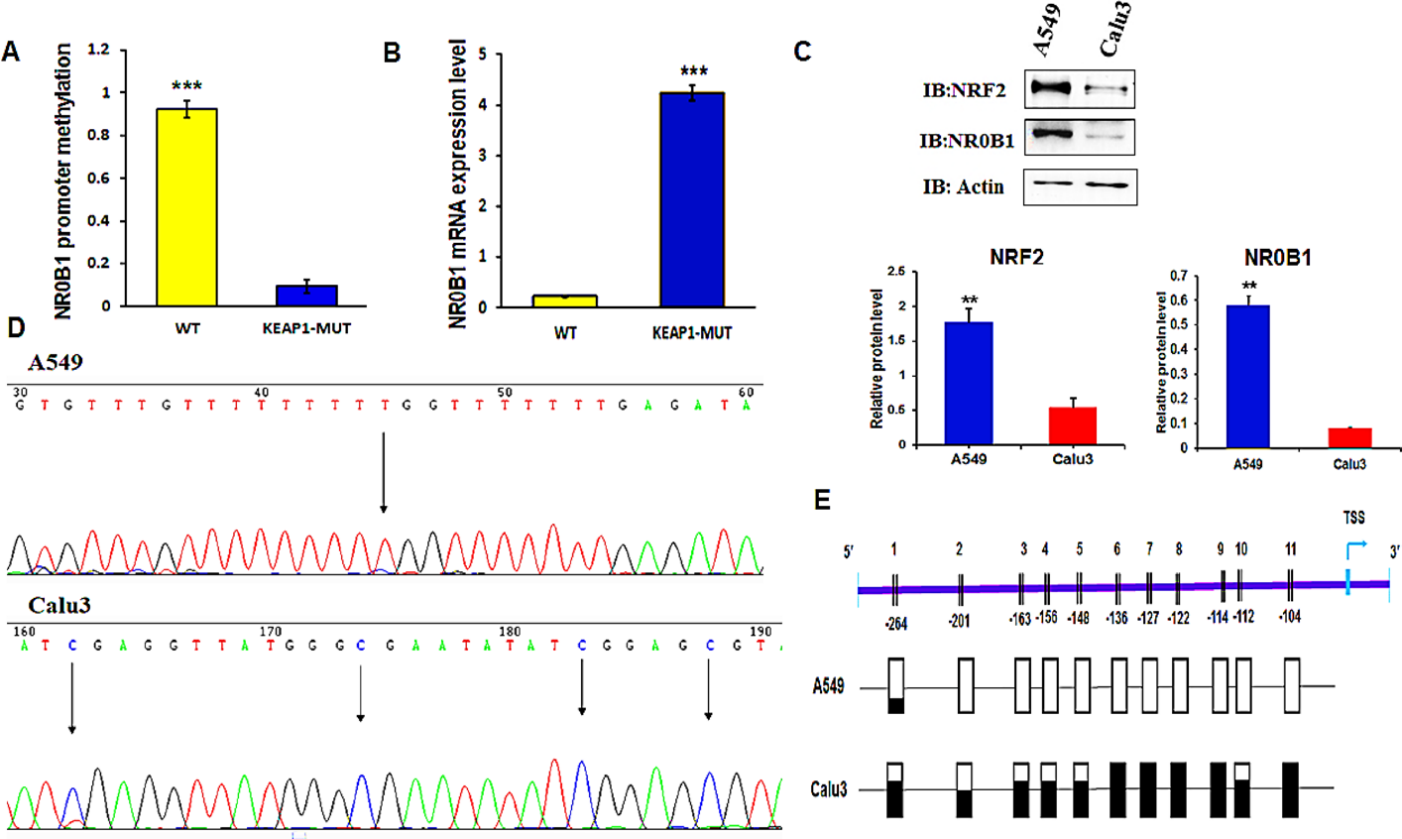
DNA methylation status of *NR0B1* promoter and *NR0B1* gene expression in LUAD cell lines. **A** Bar chart showing the differential methylation of *NR0B1* promoter between *KEAP1*-muated LUAD cell lines (n = 4) and an LUAD cell lines (n = 4) with neither *KEAP1* nor NRF2 mutations (wild-type). (*p < 0.05, ** *p* < 0.01, *** *p* < 0.001). **B** Bar chart showing the differential expression of *NR0B1* gene between *KEAP1*-muated LUAD cell lines group and wild type LUAD cell lines group. (*p < 0.05, ** *p* < 0.01, *** *p* < 0.001). **C** Western blotting analysis comparing NRF2 and NR0B1 protein levels between *KEAP1*- mutant and wild-type (WT) LUAD cell lines. NRF2 and NR0B1 were highly abundant in *KEAP1*- mutant cell line (A549) compared to the wild-type cell line (Calu3). Actin was used for normalization of loading samples. **D** A chromatogram showing the sequence of bisulfite converted DNA obtained from both A549 and Calu3 LUAD cell lines. **E** Schematic diagram, representing the DNA methylation status of *NR0B1* promoter in both A549 and Calu3 cell lines by showing the positions and methylation status of 11 CpG sites in *NR0B1* promoter. Boxes represent CpG sites and black color represent degree of methylation of the CpG sites. The 11 CpG sites were almost unmethylated in A549, while highly methylated in Calu3 cell lines

First, we used CCLE to download RRBS-1Kb upstream TSS DNA methylation and RNA-seq mRNA expression data from 1019 cell lines. Using the COSMIC database, we identified human LUAD cell lines that harbored *KEAP1* mutations. We selected four LUAD cell lines with *KEAP1* mutations (H1395, A549, H2030 and H2122) as the *KEAP1-*mutant group. In addition, we selected four human LUAD cell lines that had neither *NRF2* nor *KEAP1* mutations (H358, Calu3, H2342 and H650) as the wild-type group. We then performed differential methylation analysis between the two groups. Interestingly, we found 9 CpG sites within a CpG island in the promoter of *NR0B1* that were differentially methylated between the two groups with a delta β value of −0.855 (Fig. 1A). In other words, these CpG sites were highly hypomethylated in *KEAP1*-mutant LUAD cell line group compared to their wild-type counterparts.

Furthermore, we carried out differential gene expression analysis between the two LUAD cell line groups. Interestingly, we found that *NR0B1* was highly over-expressed in *KEAP1*-mutant LUAD cell lines compared to their wild-type counterpart with a log fold change (log FC) of 4.19 (Fig. 1B). Next, we validated these findings by performing bisulfite sequencing PCR (BSP) for this NR0B1 promoter CpG island in the A549 cells (a representative of *KEAP1*- mutant LUAD cell line group) and Calu3 LUAD cell line (a representative of wild-type group). We also detected NRF2 protein levels in both cell lines to confirm KEAP1 dysfunction in A549 and normal function of KEAP1 in Calu3. Additionally, we detected NR0B1 protein levels in both cell lines. The 9 CpG sites were found unmethylated in A549 and highly methylated in Calu3 cells, which is consistent with our multi-omics data analysis. Surprisingly, we found two other CpG sites within the same CpG island that showed unmethylation in A549 cells, but hypermethylation in the Calu3 cells (Fig. 1D, E). Moreover, NRF2 protein levels were significantly higher in A549 cells compared to the markedly lower levels observed in Calu3 cells. This difference confirms the presence of destabilizing KEAP1 mutations in A549 cells, which impair KEAP1 function and consequently enhance NRF2 activity. In consistent with the dry data, NR0B1 protein level was much higher in the A549 than its level in Calu3 cells (Fig. 1C).

### NR0B1 is hypomethylated in KEAP1-mutant TCGA- LUAD patients

**Fig. 2:**
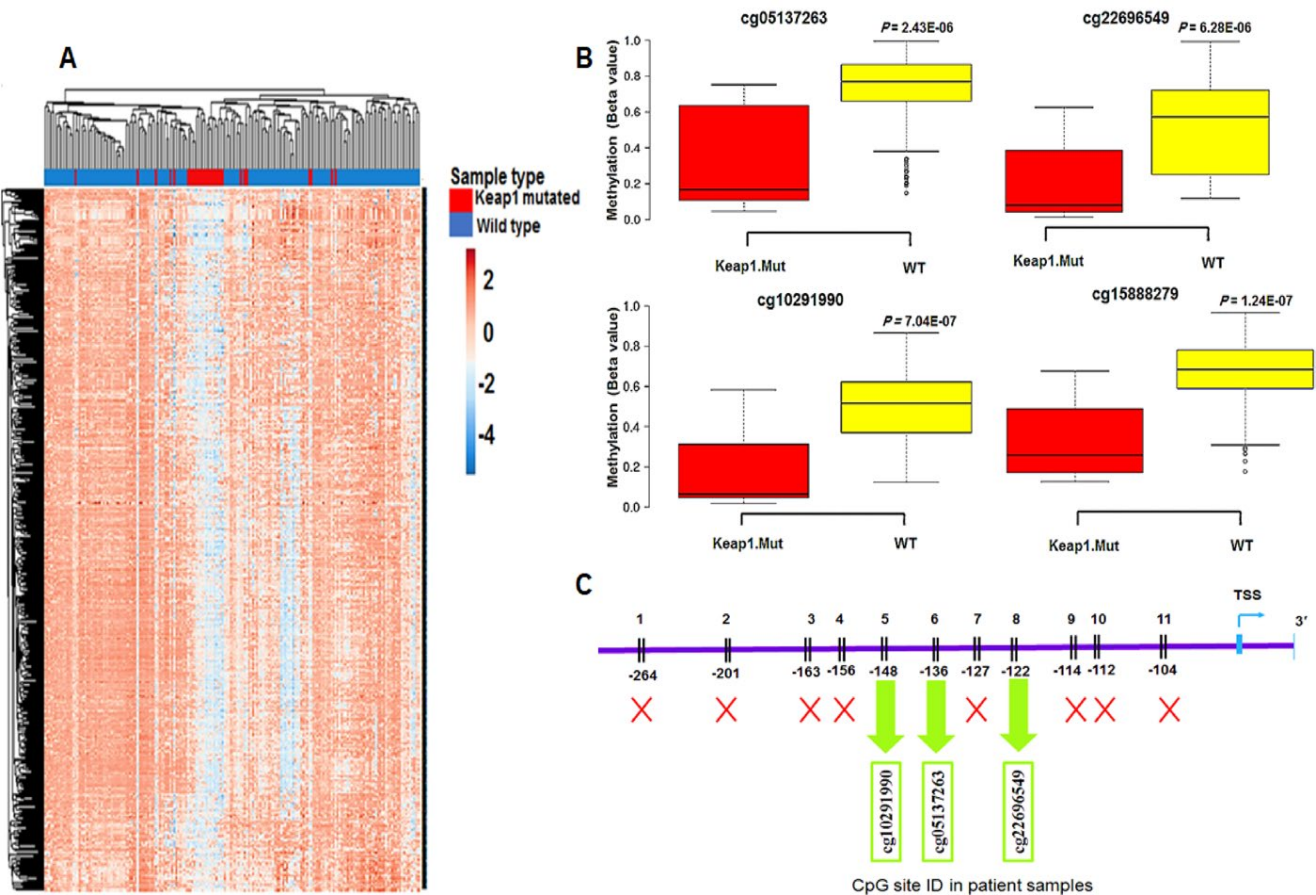
DNA methylation status of *NR0B1* promoter in *KEAP1*-mutant LUAD patient samples. **A** Heatmap showing the differentially-methylated CpG sites in *KEAP1*-mutant LUAD patients compared to WT (delta β >|0.2|, *FDR* < 0.05). Methylation Beta-values are represented as row Z-score. The blue color indicates decreased CpG methylation while the pink color indicates increased CpG methylation. **B** Box plots showing the differential methylation of 4 CpG sites located in the promoter of *NR0B1* between *KEAP1*-mutant and wild-type LUAD tumor samples. (WT, wild-type; *KEAP1*.Mut, *KEAP1*-mutant). **C** Schematic diagram showing the hypomethylated CpG sites of *NR0B1* promoter in *KEAP1*-mutant patient samples in relation to the 11 hypomethylated CpG sites in *KEAP1*-mutant cell lines. Only 3 of the 11 hypomethylated CpG sites in *KEAP1*-mutant cell lines were also found hypomethylated in *KEAP1*-mutant patient samples. Green arrows were used to show these 3 CpG sites

We found a CpG island located in the promoter of *NR0B1* gene that showed hypomethylation in *KEAP1-*mutant compared to wild-type LUAD cell lines. Correspondingly, *NR0B1* was overexpressed in *KEAP1-*mutant LUAD cell lines compared to their wild-type counterparts. In order to investigate these findings in LUAD patient samples, we stratified TCGA-LUAD patient samples (*n* = 478) into two groups: *KEAP1-*mutant group (83 patients) and wild-type group (395 patients). We then subjected the two groups to differential DNA methylation analysis (Fig. 2A). We set |0.2| as a cutoff value for delta beta (delta β) and FDR < 0.05 to consider significant differential methylation of the CpG sites between the two groups. Interestingly, we found 636 differentially methylated CpG sites between the *KEAP1*-mutant and wild-type groups. A total of 592 CpG sites were hypomethylated, while 44 CpG sites were hypermethylated, confirming the results of our previous study which showed that *KEAP1* mutations were associated with DNA hypomethylation in LUAD (Additional file 1: Table S1). Furthermore, 4 CpG sites in the promoter region of *NR0B1* were hypomethylated in the *KEAP1-*mutant group compared to the wild-type group (Fig. 2B). Surprisingly, 3 of these 4 CpG sites were found in the same CpG island of *NR0B1* promoter, which was previously detected hypomethylated in the *KEAP1-*mutant LUAD cell lines (Fig. 2C). These 3 CpG sites cg05137263, cg22696549, and cg10291990 were located in a CpG island which is 200 bp upstream of the TSS with delta β values of −0.39, −0.30, and − 0.31, respectively. The fourth CpG site, cg15888279 was N-Shore and located within 1500 bp upstream the TSS with a delta β value of − 0.34.

### NR0B1 is overexpressed in KEAP1-mutant TCGA LUAD patients

**Fig. 3:**
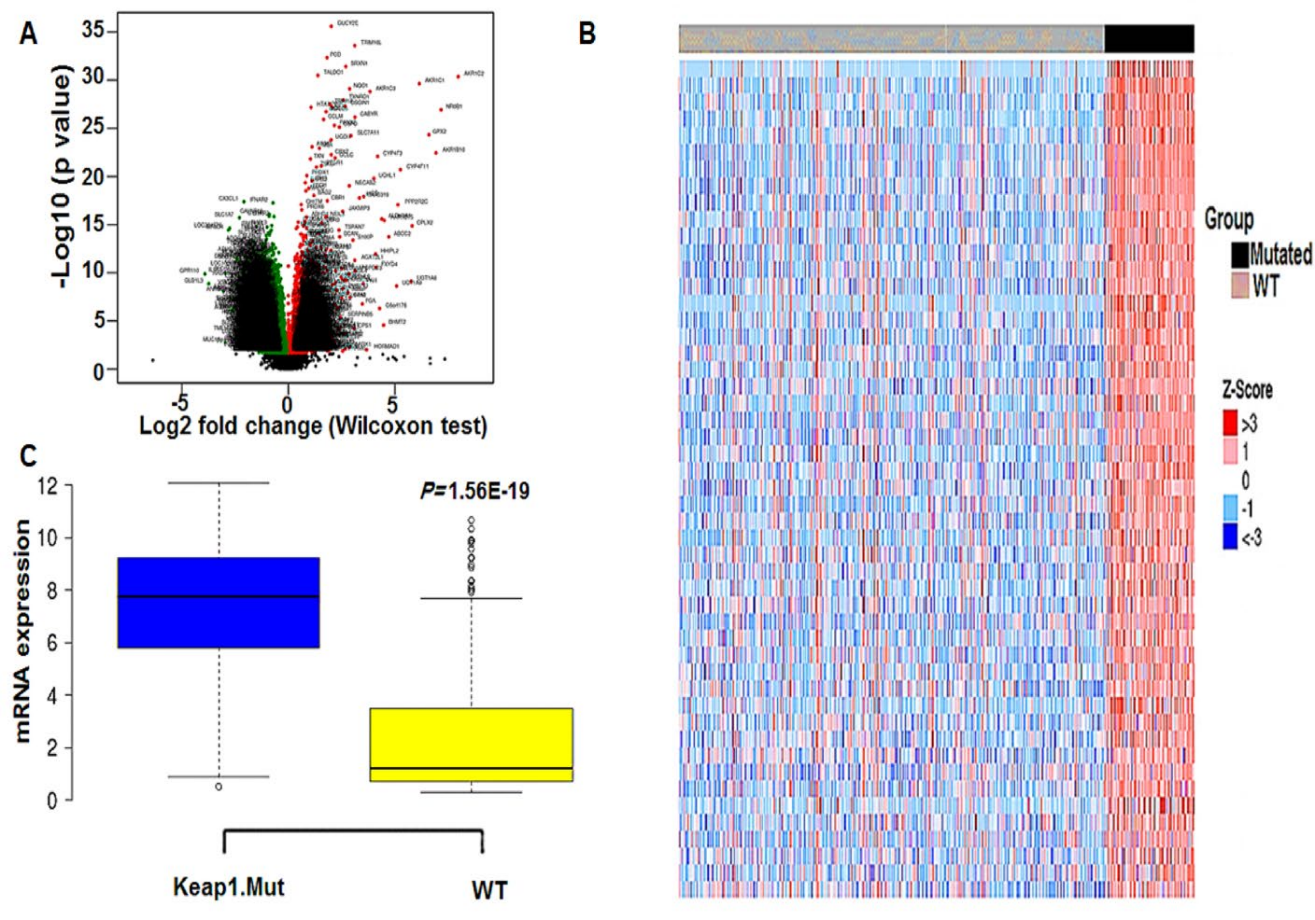
*NR0B1* gene expression in *KEAP1*-mutant LUAD patient samples. **A** Volcano plot showing the distribution of DEGs between *KEAP1*-mutant and wild-type LUAD patient samples based on significance and fold change. **B** Heatmap showing the top up-regulated genes between *KEAP1***-**mutant and wild**-**type LUAD patient samples with Log Fc > 1.5 and FDR < 0.05. **C** Box plot showing the differential expression of *NR0B1* between *KEAP1*-mutant and wild-type LUAD tumor samples. (WT, wild-type; *KEAP1*.Mut, *KEAP1*-mutant)

In order to investigate *NR0B1* gene expression in LUAD patients with *KEAP1* mutations compared to wild-type counterparts, we performed differential gene expression analysis between *KEAP1*-mutant (*n* = 83) and wild-type (*n* = 395) groups. The analysis resulted in 19,984 differentially expressed genes (DEGs), with only 4,742 of them being statistically significant DEGs (FDR < 0.05) (Fig. 3A). We only considered DEGs with log FC > 1.5, and FDR < 0.05 (Fig. 3B).

**Fig. 4:**
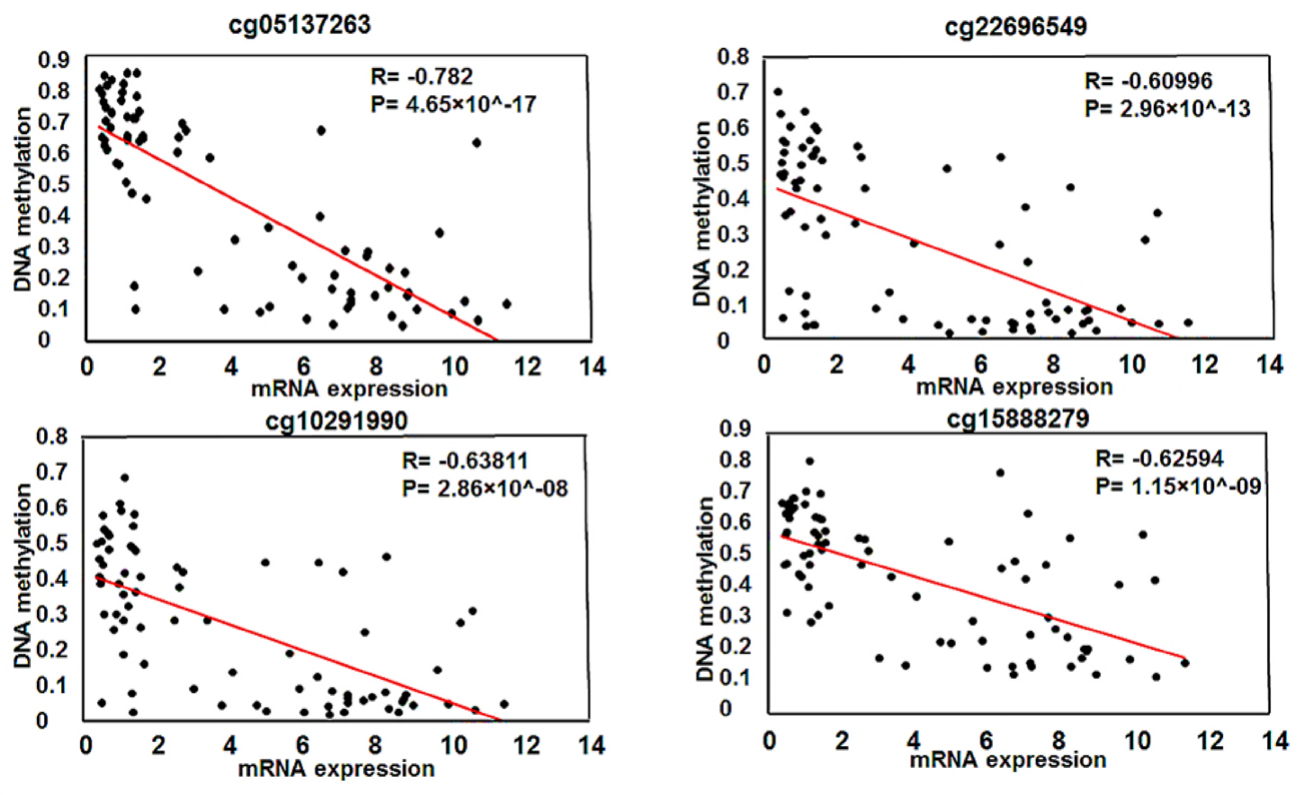
Correlation analysis between DNA methylation and gene expression. Scatter plots showing the Spearman’s correlation between the DNA methylation of the 4 CpG sites and *NR0B1* expression. A strong negative correlation can be seen between the methylation of the 4 CpG sites and *NR0B1* expression

With these criteria we had a total of 252 DEGs with 138 genes were overexpressed while 114 genes were down-regulated (Additional file 2: Table S1). *NR0B1* was found highly over-expressed in the *KEAP1*-mutant compared to wild-type LUAD patient samples with a log FC = 7.17, coming in the third place in the list of the overexpressed genes in *KEAP1*-mutant samples (Fig. 3C). Further, we investigated the role of the identified differentially methylated CpG sites in the promoter of *NR0B1* in regulating the expression of *NR0B1*. This was accomplished by performing Pearson’s correlation analysis on the methylation beta values of the 4 differentially methylated CpG sites in the *NR0B1* promoter with the corresponding *NR0B1* mRNA expression values across 77 LUAD patient samples. As mentioned earlier, cg05137263, cg22696549 and cg10291990 were in a CpG island located 200 bp upstream of the TSS, whereas cg15888279 was located 1500 bp upstream the TSS. Interestingly, a strong negative correlation was detected between the methylation status of the four CpG sites and *NR0B1* gene expression. The four CpG sites cg05137263, cg22696549, cg10291990, and cg15888279 had statistically significant negative Pearson’s correlation R values of −0.782, −0.609, −0.638, and −0.626, respectively (Fig. 4). This data is consistent with previous reports, showing that the methylation status of the promoter CpG sites is inversely correlated with the expression of their corresponding gene [23]. These findings indicate that the methylation status of these 4 promoter CpG sites plays a major role in inducing the expression of *NR0B1* gene in *KEAP1*-mutant LUAD patient samples. Recently, *NR0B1* has been suggested as NRF2 target. It is worth noting that the ultimate effect of *KEAP1* mutants is the enhanced activity of NRF2 and consequently the overexpression of NRF2 targets. Therefore, it wasn’t surprising to find that *NR0B1* was overexpressed in *KEAP1*-mutant LUAD patient samples. However, our data suggest that the increased binding of NRF2 to its binding site in the promoter of *NR0B1* may not be the sole mechanism that is initiated by *KEAP1* mutations which eventually leads to *NR0B1* over-expression in LUAD patient samples. Here, we demonstrated that *KEAP1* mutations are associated with a change in the methylation status of several CpG sites in the *NR0B1 promoter,* which may play a major role in driving *NR0B1* overexpression in *KEAP1*-mutatnt LUAD patient samples.

### Hypomethylated NR0B1 promoter and NR0B1 overexpression are associated with lower survival rates in patients with LUAD

**Fig. 5:**
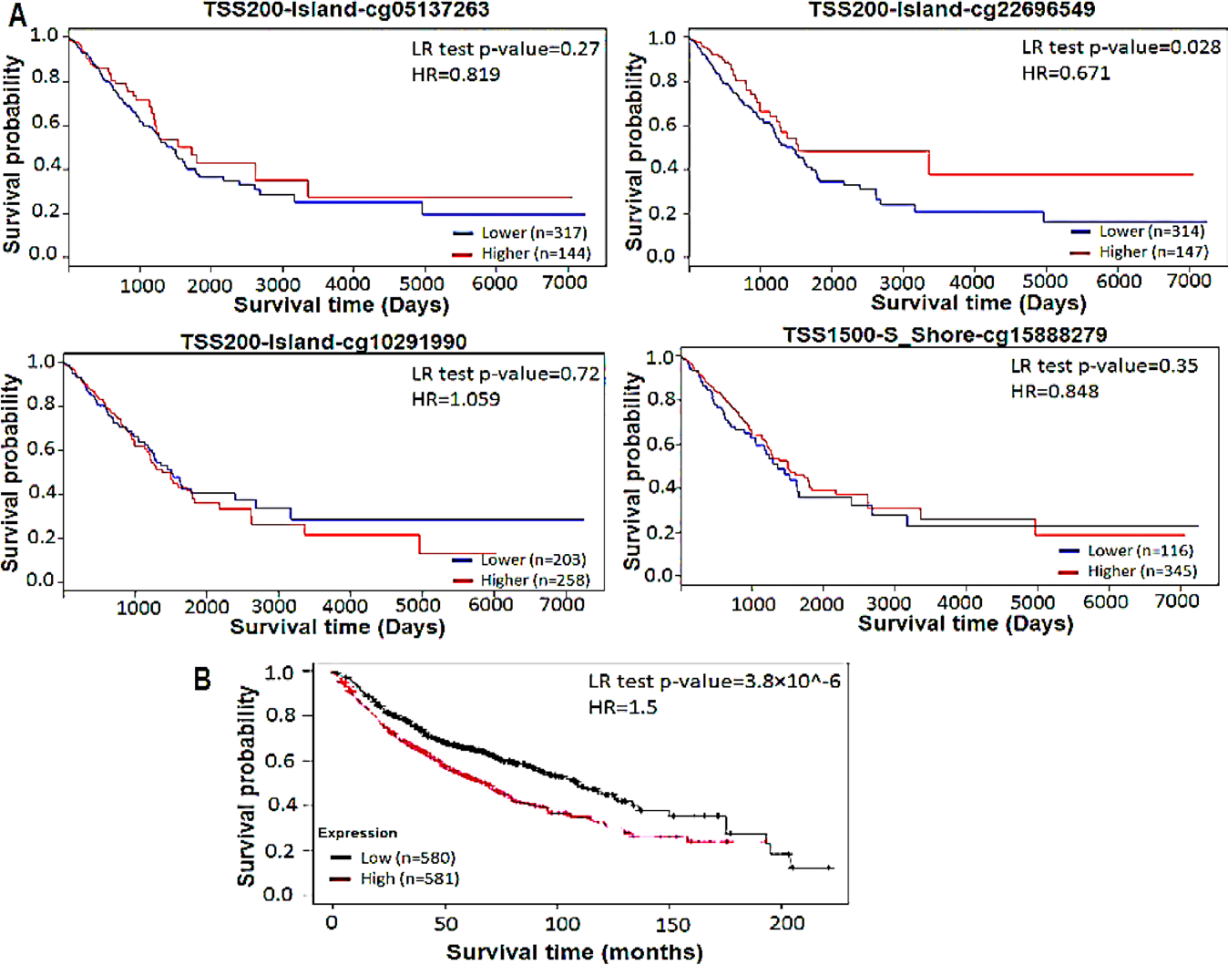
Survival analysis to predict prognosis outcome. **A** Kaplan-Meier overall survival plots showing that the methylation status of cg05137263, cg10291990, and cg15888279 couldn’t significantly predict the survival of TCGA-LUAD patients. However, the hypomethylation of cg22696549 in the promoter of *NROB1* was associated with poor survival of TCGA-LUAD patients. **B** Kaplan-Meier survival plot showing that the over-expression of *NR0B1* was associated with poor survival of LUAD patients

We evaluated the prognostic power of the four *NR0B1* promoter CpG sites that showed hypomethylation in *KEAP1*-mutant LUAD patient samples by analyzing the overall survival in the TCGA-LUAD cohort using the Methsurv database. A total of 461 patient samples were divided into two groups for each CpG site. The first group included LUAD patients where the CpG site showed hypomethylation while the second group included LUAD patients where the CpG site showed hypermethylation. There was not a statistically significant difference between the two groups in terms of overall survival based on the methylation status of the three CpG sites, TSS200-Island-cg05137263, TSS200-Island-cg10291990, and TSS1500-S_Shore-cg15888279, with log-rank (LR) test p-value of 0.27, 0.72, and 0.3, respectively. However, TSS200-Island-cg22696549 CpG site could significantly differentiate the overall survival between the two groups, with a LR test p-value of 0.028 and hazard ratio (HR) of 0.671 (95% confidence interval [CI]: 0.464 – 9.7). The patients group (314 patients) with hypomethylated cg22696549 CpG site had lower overall survival probability than the group (147 patients) with hypermethylated cg22696549 CpG site. This indicates that the hypomethylation of cg22696549 CpG site (*NR0B1* promoter) was associated with lower overall survival rates in patients with LUAD (Fig. 5A). Therefore, the methylation status of cg22696549 CpG site can be used as a prognostic and predictive biomarker for patients with LUAD. We showed earlier that *KEAP1* mutations in LUAD were associated with the hypomethylation of cg22696549 and the other three CpG sites located in the promoter of *NR0B1*, leading to the over-expression of *NR0B1* in synergy with NRF2 activation. Thus, we evaluated the prognostic value of *NR0B1* gene expression in LUAD patients using Kaplan-Meier plotter data base. A total of 1161 LUAD patients were divided into two groups. The first group included patients with low *NR0B1* expression, while the other group had patients with high *NR0B1* expression. The patients with high *NR0B1* expressions had a lower overall survival probability (median survival time of 68 months) than the patients with low *NR0B1* expression (median survival time of 108.97 months) with significant LR test p-value of 3.8 × 10^−6 and HR of 1.5 (CI: 1.26–1.78). Thus, high *NR0B1* gene expression was associated with lower overall survival rates in LUAD patients (Fig. 5B).

**Fig. 6:**
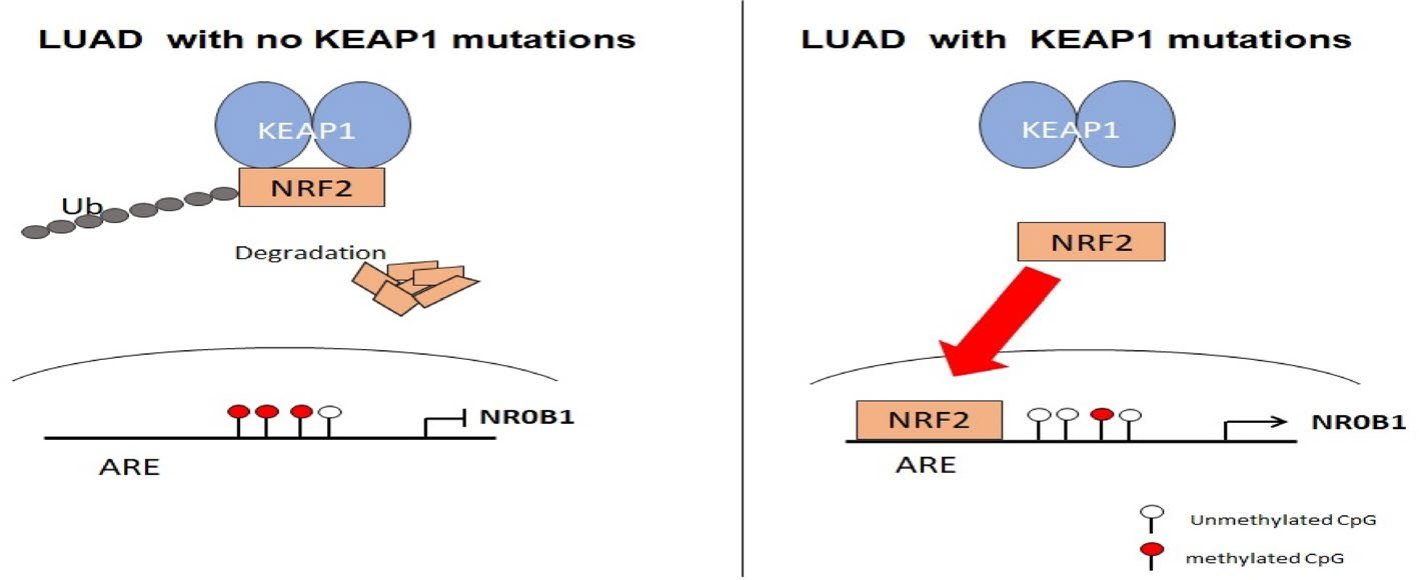
Proposed model for NROB1 expression in LUAD

## Discussion

Oda et al., have shown that reducing *NR0B1* expression in LUAD cell lines induces vulnerability to anti-cancer drugs and decreased invasion abilities [24]. Moreover, Bar-Peled et al. demonstrated that *NR0B1*, which is a strictly NRF2-regulated protein, supporting the anchorage-independent growth of *KEAP1*-mutant NSCLC cells forms a multimeric transcriptional complex important for regulating a subset of the NRF2 gene expression program in NSCLC cells [7]. Additionally, they showed that targeting a conserved cysteine residue (C274) in *NR0B1* disrupt *NR0B1* complexes and impair the anchorage-independent growth of *KEAP1*-mutant cancer cells [7]. In consistent with the finding of Bar-Peled et al., we found that *NR0B1* was highly overexpressed in *KEAP1*-mutant LUAD cell lines and patient samples compared to their wild-type counterparts. In our previous study, we showed that several genes, including well-known NRF2 targets were hypomethylated in *KEAP1*-mutant LUAD patient samples. Furthermore, a strong correlation between the hypomethylated CpG sites and the expression of their corresponding genes was observed. This indicates the presence of a potential synergy between DNA methylation alterations associated with *KEAP1* mutations and the transcription factor NRF2 that may lead to the overexpression of some NRF2 target genes.

Consistent with the findings of our previous study [8], we showed that several CpG sites within the promoter of *NR0B1* were hypomethylated in the *KEAP1*-mutant LUAD cell lines and patient samples compared to their wild-type counterparts. Additionally, there was a strong correlation between the methylation status of these CpG sites and the expression of *NR0B1*. In agreement with our findings, it has been reported that *NR0B1* expression in clinical samples is inversely correlated with the proportion of methylated CpG sequences around the transcription initiation site of the *NR0B1* gene, suggesting the presence of epigenetic control of *NR0B1* transcription in LUAD [25].

*KEAP1* mutations in LUAD result in higher activity of NRF2 and the subsequent overexpression of its downstream genes that play a major role in reducing cellular oxidative stress. This might give an explanation of the observed DNA hypomethylation of *NR0B1* promoter in *KEAP1*-mutant LUAD cell lines and patient samples since it has been reported that DNA—methyltransferases activity, including DNA methyltransferase 1 (DNMT1) and DNMT3B is largely affected by cellular redox status [26].

Furthermore, we propose cg22696549—one of the hypomethylated CpG sites in the *NR0B1* promoter in *KEAP1*-mutant LUAD patient samples—as a potential biomarker for LUAD, given that the hypomethylation of cg22696549 was associated with poor survival of LUAD patients. Additionally, we demonstrated that the over-expression of *NR0B1* was associated with lower survival rates in 1161 LUAD patients.

While our findings suggest that *KEAP1* mutations and subsequent NRF2 activation contribute to *NR0B1* overexpression, it is important to recognize that *NR0B1* expression is regulated by multiple pathways beyond NRF2. Previous studies have shown that the Wnt/β-catenin signaling pathway can upregulate *NR0B1* expression [27]. Furthermore, transcription factors including SF1 (Steroidogenic Factor 1) and LRH-1 (Liver Receptor Homolog-1) have been reported to directly regulate *NR0B1* expression in certain cancer types, adding further complexity to its regulation [28]. Moreover, in Ewing’s sarcoma, the EWS/FLI fusion protein directly upregulates *NR0B1*, highlighting the role of alternative oncogenic transcription factors in its regulation [29]. Additionally, NR0B1 has been implicated in modulating ferroptosis resistance through activation of the c-JUN/NRF2-CBS signaling axis in lung cancer cells, suggesting a complex interplay between NR0B1 and multiple transcriptional regulators [30]. Therefore, we acknowledge that the regulatory landscape of *NR0B1* is multifactorial, and further studies are warranted to dissect these alternative regulatory mechanisms in the context of *KEAP1*-mutant lung adenocarcinoma. In conclusion, we demonstrated that the *NR0B1* promoter is hypomethylated in *KEAP1*-mutant LUAD cell lines and patient samples. Additionally, we found that *NR0B1* was overexpressed in *KEAP1*-mutant LUAD cell lines and patient samples with an observed strong negative correlation between the methylation status of the hypomethylated CpG sites in *KEAP1*-mutant LUAD and the expression of *NR0B1.* These findings suggest a potential synergy between *KEAP1* mutation-associated DNA methylation alterations (in the *NR0B1* promoter*)* and the transcription factor NRF2, which may drive *NR0B1* overexpression. Furthermore, we identified that both the methylation status of the cg22696549 CpG site (in the *NR0B1* promoter) and the *NROB1* expression levels can predict the overall survival of LUAD patients (Fig. 6).

## Supplementary Information

Supplementary Material 1.

Supplementary Material 2.

Supplementary Material 3.

## Data Availability

The bisulfite sequencing data supporting the findings of this study have been deposited in the Zenodo repository and are publicly available at: [https://zenodo.org/records/16907360] with DOI [https://doi.org/10.5281/zenodo.16907360]. Publicly available datasets from The Cancer Genome Atlas (TCGA) and the Cancer Cell Line Encyclopedia (CCLE) were also used in this study. TCGA data were retrieved from the Genomic Data Commons (GDC) portal (https://portal.gdc.cancer.gov), and CCLE data were obtained from the Broad Institute (https://portals.broadinstitute.org/ccle).
