# Supplementary material for: Aberrant DNA methylation and overexpression of NR0B1 are prognostic biomarkers in KEAP1-mutant lung adenocarcinomas
Source: Discov Oncol. 2025 Aug 27;16:1641. doi: 10.1007/s12672-025-03484-1 (PMC12391575; doi:10.1007/s12672-025-03484-1)
Supplement: Supplementary file 3 — Supplementary Material 3. [file 12672_2025_3484_MOESM3_ESM.pptx]

## Slide 1
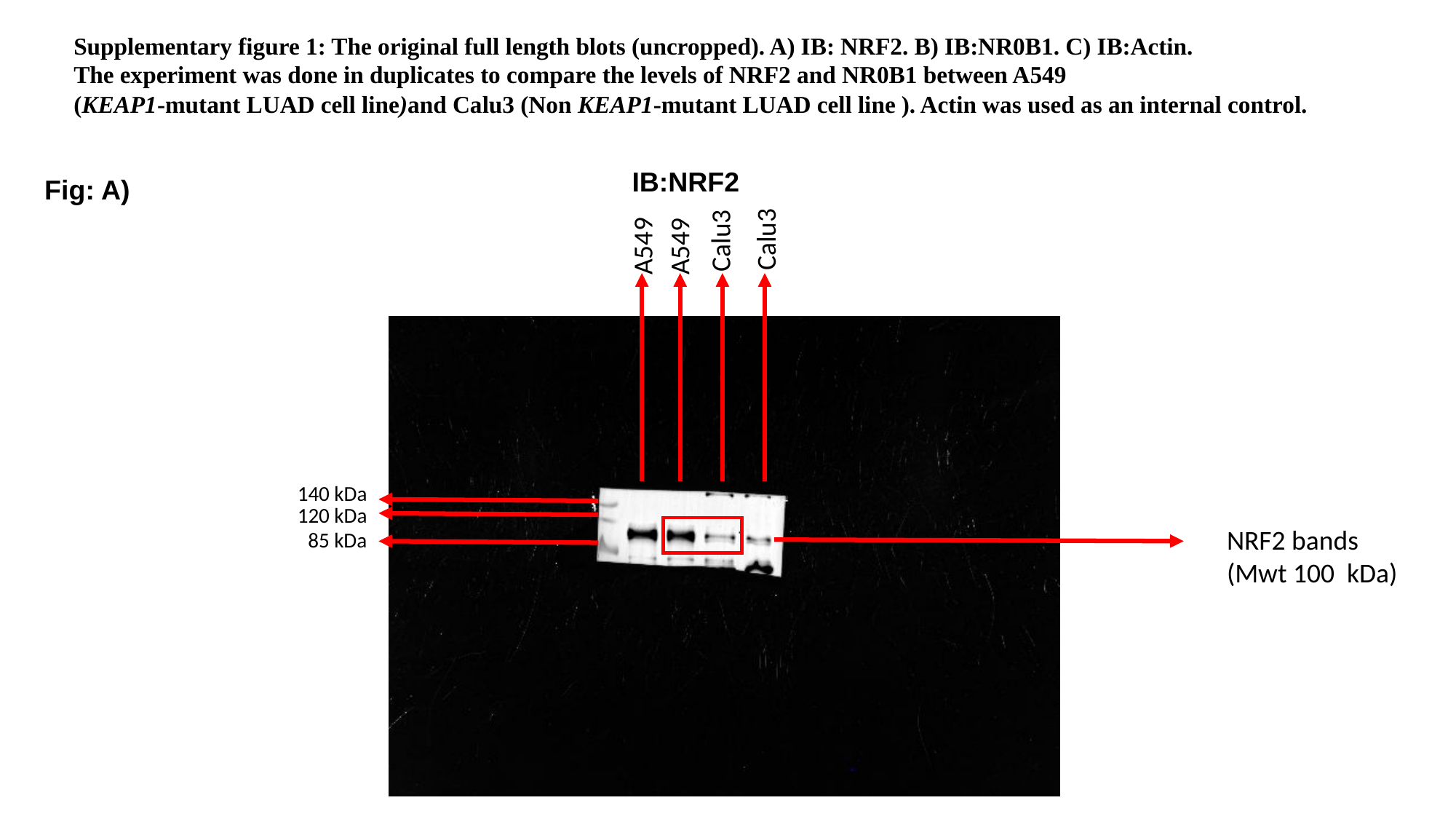

Supplementary figure 1: The original full length blots (uncropped). A) IB: NRF2. B) IB:NR0B1. C) IB:Actin.
The experiment was done in duplicates to compare the levels of NRF2 and NR0B1 between A549
(KEAP1-mutant LUAD cell line)and Calu3 (Non KEAP1-mutant LUAD cell line ). Actin was used as an internal control.
 IB:NRF2
 Fig: A)
Calu3
Calu3
A549
A549
140 kDa
120 kDa
NRF2 bands
(Mwt 100 kDa)
85 kDa

## Slide 2
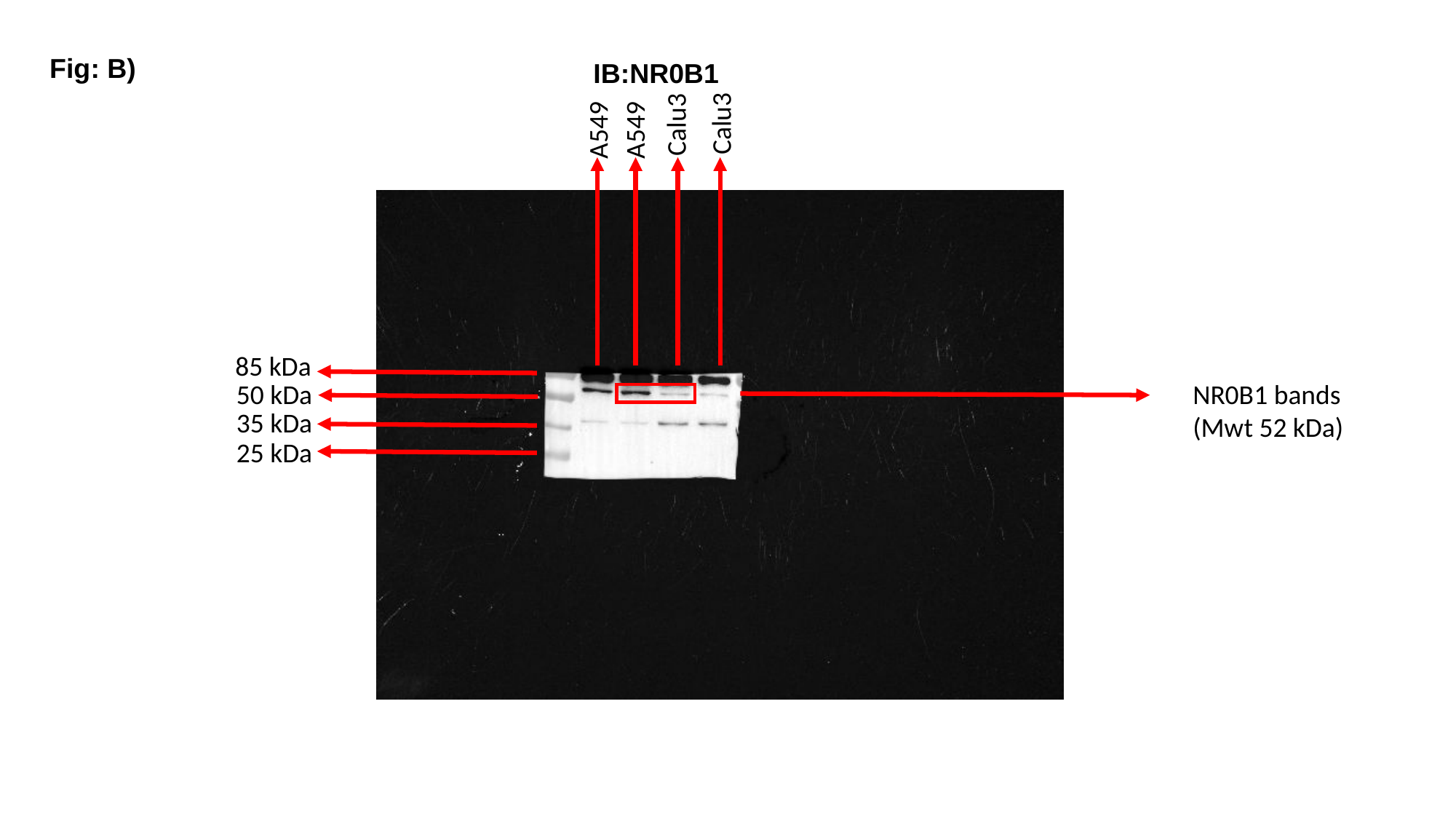

Fig: B)
IB:NR0B1
Calu3
Calu3
A549
A549
85 kDa
50 kDa
NR0B1 bands
(Mwt 52 kDa)
35 kDa
25 kDa

## Slide 3
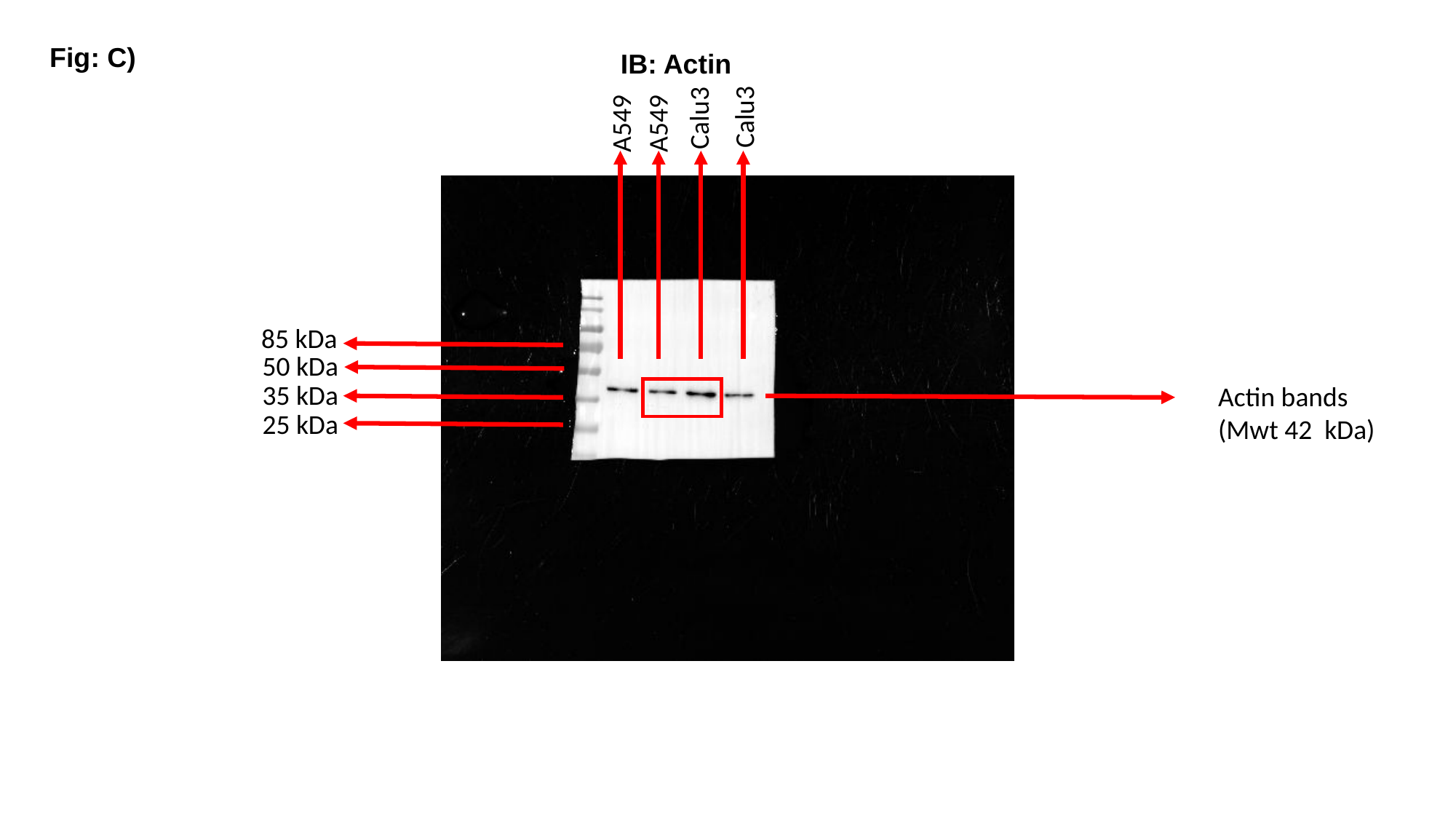

Fig: C)
IB: Actin
Calu3
Calu3
A549
A549
85 kDa
50 kDa
35 kDa
Actin bands
(Mwt 42 kDa)
25 kDa
